# Ultrasound elastography techniques for diagnosis and follow-up of hepatic veno-occlusive disease

**DOI:** 10.1038/s41409-019-0432-5

**Published:** 2019-01-24

**Authors:** Lorenzo Lazzari, Paolo Marra, Raffaella Greco, Fabio Giglio, Daniela Clerici, Elena Venturini, Pierluigi Paesano, Serena Albanese, Francesca Serio, Fabio Ciceri, Jacopo Peccatori

**Affiliations:** 1 Hematology and Bone Marrow Transplantation Unit, IRCCS San Raffaele Scientific Institute, Milano, Italy; 2 Department of Radiology, IRCCS San Raffaele Scientific Institute, Milano, Italy; 3 Vita-Salute San Raffaele University, Milano, Italy

**Keywords:** Diagnosis, Acute myeloid leukaemia

Hepatic sinusoidal obstruction syndrome/veno-occlusive disease (SOS/VOD) is a potentially life-threatening complication that can occur after hematopoietic stem cell transplantation (HSCT). Severe SOS/VOD rapidly evolve in multiple organ dysfunction syndrome (MODS), and it is associated with a mortality rate that exceeds 80% [1, 2]. Ultrasound elastography techniques, including two-dimensional shear wave elastography (2D-SWE) and transient elastography (TE), may be useful in allowing early diagnosis, thus enabling a prompt therapeutic intervention before irreversible tissue damage occur [3–5].

Liver stiffness measurement (LSM) was performed using 2D-SWE (ShearWave) and TE (FibroScan). Both of them are user-friendly, accurate, non-invasive techniques, been proven to have a high performance in diagnosing and staging liver fibrosis [6]. These techniques measure shear wave velocity in the liver, which is increased in the context of SOS/VOD because of congestion and portal hypertension [7]. Liver stiffness is expressed as a velocity which is converted in kiloPascals (kPa) through the Young modulus. Normal values range respectively from 2.6 to 6.2 kPa for 2D-SWE, and from 3.9 to 7.1 kPa for TE [8, 9]. According to guidelines, a minimum of three measurements for 2D-SWE and ten measurements for TE are required, and results are considered reliable when the interquartile range (IQR)/median ratio is lower than 30%. SOS/VOD diagnosis was made according to the European society of Bone and Marrow Transplantation (EBMT) criteria [10].

A 41-year-old man was diagnosed with FLT3-ITD-positive acute myeloid leukemia (AML) in March 2018. For hyperleukocytosis, at the time of diagnosis he underwent therapeutic leukapheresis, and he subsequently required two induction chemotherapy regimen to obtain a complete hematologic remission.

A HLA-identical allogeneic HSCT from a sibling donor was then performed at our Institute. A myeloablative (MA) conditioning regimen using intravenous Busulfan (9.6 mg/Kg) and Thiotepa (10 mg/Kg,) alongside with Fludarabine (150 mg/m^2^), was selected. Post-transplant Cyclophosphamide (100 mg/Kg) and Rapamycin were used for graft-versus-host disease (GVHD) prophylaxis [11]. No LSM data were available before HSCT. Pre-transplant abdominal ultrasound revealed only a mild steatosis, and liver biochemical analysis were in the therapeutic range. Risk factors for SOS/VOD in our patient were the use of a MA conditioning with double alkylating agents, specifically the combination of high-dose Busulfan and Thiotepa, and fungal prophylaxis with an azole [10].

**Fig. 1:**
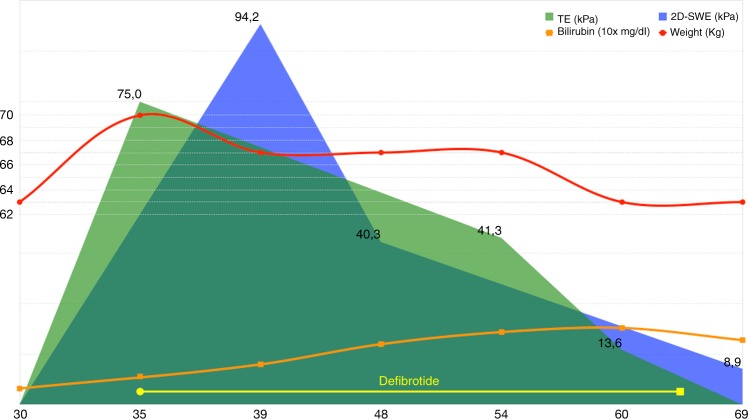
Clinical, laboratory, and instrumental response after Defibrotide therapy in a patient with VOD. On the x-axis days from HSCT. In blue 2D-SWE results (two-dimensional shear wave elastography), in green TE (transient elastography), red line for patient’s weight, orange line for total bilirubin levels, yellow bar for defibrotide administration

At day +32 from HSCT infusion, the patient began developing painful hepatomegaly, ascites and rapid weight increment (≥10% at diagnosis) despite an optimal diuretic therapy. An abdominal ultrasound showed only nonspecific signs. The main hepatotropic virus tested were negative, and a mild increase in transaminases and liver stasis indexes occurred. Conversely, serum bilirubin was in the normal range. No signs of GVHD were present and Rapamycin has always been kept in the normal range. On day +35 a TE analysis was found to be definitely abnormal with markedly increased liver stiffness at 75 kPa (IQR/median ratio 2%) and a diagnosis of late-onset severe hepatic SOS/VOD was made. Defibrotide 25 mg/Kg/die was started immediately after diagnosis. Furthermore, a 2D-SWE performed on day +41 revealed a value of 94.2 kPa, confirming the other elastography datum (Fig. 1).

TE and 2D-SWE were used weekly to assess response during treatment, showing a progressive decrease in liver stiffness. 2D-SWE performed on day +48 and +69 was 40.3 and 8.9 kPa, respectively; TE performed on day +54 and +60 was 41.6 and 13.6 kPa, respectively. A rise in serum bilirubin appeared only later during the course of the disease, peaking on day +59 (2.1 mg/dl). Defibrotide was continued until day +66, time when clinical and LSM, but not laboratory, data confirmed a trend to remission (Fig. 1). No severe adverse effects occurred during the course of the therapy. LSM continued to improve and returned to normal values within 100 days from HSCT.

SOS/VOD is a clinical syndrome characterized by painful hepatomegaly, ascites, weight gain, and jaundice, occurring more frequently in the first weeks after HSCT. The average incidence of this complication ranges from 5% to 15%. The most severe forms are associated with a mortality rate that exceeds 80% in untreated patients [1]. Principal risk factors for this condition include the type of transplant, the intensity of the conditioning regimen, and pre-transplant liver dysfunction [10]. Other conditions may mimic SOS/VOD in the early post-transplant period, like hepatic GVHD, making sometimes arduous to achieve a precise diagnosis.

An early detection of SOS/VOD should be a priority, given the presence of a drug, Defibrotide, that has been shown to be an effective treatment option [4]. Moreover, a higher survival rate was seen when therapy with Defibrotide was initiated earlier in the disease course [5]. Unfortunately, current diagnosis criteria, the Baltimore [12] and the modified Seattle criteria [13], have limited sensitivity and specificity, and they are able to identify severe forms only when serious organ damage or MODS has already occurred. Furthermore, these criteria do not contemplate a diagnosis of late-onset SOS/VOD, a condition in which hyperbilirubinemia could be delayed or completely absent. Consequently, an EBMT panel proposed new criteria for the diagnosis of this condition in adult patients [10], incorporating sonography techniques as a tool for helping in the diagnosis when only weight gain, ascites or painful hepatomegaly are present.

Standard ultrasound imaging may be helpful in the differential diagnosis in patients with suspected SOS/VOD. Liver biopsy and hemodynamical studies are considered gold standard, but they are invasive and should be reserved for patients in whom the diagnosis of SOS/VOD is unclear. LSM using ultrasound elastography techniques could be useful in this scenario, particularly in facilitating early diagnosis. Several ultrasound elastography techniques using different excitation methods have been developed. These can be classified into strain imaging methods that use internal or external compression stimuli, and shear wave imaging based on ultrasound-generated traveling shear wave stimuli, like 2D-SWE and TE [14]. They are easy-learning, accurate, non-invasive, and relatively low-cost instruments, principally validated for the diagnosis and staging of liver fibrosis [7]. 2D-SWE is available on most new generation US scanner and has the advantage, over Fibroscan, of combining US morphologic imaging with elastography: this allows the choice of the best acoustic window to acquire an accurate liver elastogram. As we saw in our patient’s case, liver stiffness data obtained using 2D-SWE and TE are superimposable, and both techniques can be effectively employed in SOS/VOD diagnosis and follow-up.

Only a few single-center studies experimented on the use of LSM techniques in the SOS/VOD scenario. Preliminary results agree that a sudden increase in LSM values during follow-up precedes a diagnosis of SOS/VOD, and that elastographic alterations appear earlier than clinical and biochemical signs [15]. Our patient developed a late-onset SOS/VOD without biochemical data suggesting the underlying condition, and only the clinical picture supported by liver stiffness values allowed us to start Defibrotide before the appearance of organ impairment signs. Shear wave analysis also permitted us to follow the patient during the treatment phase, showing the right time to stop the therapy as opposed to biochemical data. Following the patients during the pre- and post-transplant procedure with serial LSM scans may reveal those that are more prone to develop this potentially fatal condition, and allows a more effective therapeutic intervention with Defibrotide.

In conclusion, future perspectives should concentrate in implementing ultrasound elastography techniques in routine clinical practice, especially for patients considered at high risk for this life-threatening complication. Multicenter prospective clinical trials could better evaluate and validate the use of LSM techniques in the diagnosis and follow-up of SOS/VOD.
